# Selection for environmental variance of litter size in rabbits involves genes in pathways controlling animal resilience

**DOI:** 10.1186/s12711-021-00653-y

**Published:** 2021-07-13

**Authors:** Cristina Casto-Rebollo, María José Argente, María Luz García, Agustín Blasco, Noelia Ibáñez-Escriche

**Affiliations:** 1 Institute for Animal Science and Technology, Universitat Politècnica de València, Valencia, Spain; 2 Departamento de Tecnología Agroalimentaria, Universidad Miguel Hernández de Elche, Orihuela, Spain

## Abstract

**Background:**

Environmental variance (V_E_) is partially under genetic control, which means that the V_E_ of individuals that share the same environment can differ because they have different genotypes. Previously, a divergent selection experiment for V_E_ of litter size (LS) during 13 generations in rabbit yielded a successful response and revealed differences in resilience between the divergent lines. The aim of the current study was to identify signatures of selection in these divergent lines to better understand the molecular mechanisms and pathways that control V_E_ of LS and animal resilience. Three methods (F_ST_, ROH and varLD) were used to identify signatures of selection in a set of 473 genotypes from these rabbit lines (377) and a base population (96). A whole-genome sequencing (WGS) analysis was performed on 54 animals to detect genes with functional mutations.

**Results:**

By combining signatures of selection and WGS data, we detected 373 genes with functional mutations in their transcription units, among which 111 had functions related to the immune system, stress response, reproduction and embryo development, and/or carbohydrate and lipid metabolism. The genes *TTC23L*, *FBXL20*, *GHDC*, *ENSOCUG00000031631*, *SLC18A1*, *CD300LG*, *MC2R*, and *ENSOCUG00000006264* were particularly relevant, since each one carried a functional mutation that was fixed in one of the rabbit lines and absent in the other line. In the 3ʹUTR region of the *MC2R* and *ENSOCUG00000006264* genes, we detected a novel insertion/deletion (INDEL) variant.

**Conclusions:**

Our findings provide further evidence in favour of V_E_ as a measure of animal resilience. Signatures of selection were identified for V_E_ of LS in genes that have a functional mutation in their transcription units and are mostly implicated in the immune response and stress response pathways. However, the real implications of these genes for V_E_ and animal resilience will need to be assessed through functional analyses.

**Supplementary Information:**

The online version contains supplementary material available at 10.1186/s12711-021-00653-y.

## Background

The environmental variance (V_E_) of a trait is the within-individual variation of the phenotypic values of that trait due to environmental factors [1, 2]. V_E_ is partially under genetic control, which means that individuals sharing the same environment can have different V_E_ because they have different genotypes [1]. Indeed, there have been successful divergent selection experiments for V_E_ in mice [3] and rabbits [4]. V_E_ was recently proposed as a measure of animal resilience [5], which has been defined as an animal’s ability to cope with environmental disturbances and the rapid recovery of its productive performance [6, 7]. Differences in resilience have been reported in rabbit lines that were divergently selected for high and low V_E_ of litter size (LS), with the line with a low V_E_ of LS being more resilient [8]. According to Colditz and Hine [7], the immune system, nervous system and cell receptors are essential for modulating animal resilience and allowing detection of and response to environmental perturbations, such as pathogen infections.

Genome-wide associations studies (GWAS) for V_E_ in livestock have identified candidate genes that are involved in the immune response, which boosts the inflammatory response [9–11]. In rabbit lines that were divergently selected for V_E_ of LS, Casto-Rebollo et al. [11] identified functional mutations in candidate genes that are involved in the immune system, the nervous system, and the development of sensory structures. However, V_E_ is a complex trait with a low heritability [12] and low phenotype accuracy, which makes the identification of all the loci that affect V_E_ by GWAS only, difficult [13]. Analyses of signatures of selection, which do not require phenotype data, could help to identify more loci that affect V_E_ of LS. Several methods for the detection of signatures of selection have been proposed and are based on different assumptions according to the pattern of positive selection to be detected [14]: (1) reduction of the local genomic variability, (2) modification of the spectrums of allele frequency, or (3) variation of the linkage disequilibrium (LD). However, because of these different assumptions, the three methods are not strongly correlated [15, 16] and, thus, have to be used in conjunction to identify the largest possible number of signatures of selection.

The aim of this study was to identify signatures of selection in rabbits that were divergently selected for high and low V_E_ of LS during 13 generations [4], in order to determine the molecular mechanisms and pathways that control the V_E_ of LS and animal resilience. We used three methods to identify signatures of selection in combination with whole-genome sequencing (WGS) analysis to highlight the genes with functional mutations. This study complements a previous GWAS for V_E_ of LS using the same rabbit lines [11].

## Methods

### Animals and genotyping data

The rabbits used in this study were from generations 11 and 13 and from the base population of a divergent selection experiment for high and low V_E_ of LS that was carried out at the University Miguel Hernández in Elche, Spain [4]. In total, 473 genotypes were used from 96 does from the base population, 282 from generation 11 (147 from the line with high V_E_ of LS and 135 from the line with low V_E_ of LS), and 95 from generation 13 (46 from the line with high V_E_ of LS and 49 from the line with low V_E_ of LS). Genomic DNA was isolated from blood and tissue samples using standard protocols. Genotyping was performed with the 200 K Affymetrix Axiom Orcun Single Nucleotide Polymorphism (SNP) array (Thermo Fisher Scientific) and quality control was done with the Axiom Analysis Suite 3.1 platform from Thermo Fisher Scientific and PLINK v.1.9 software [17]. Quality control removed animals with a call rate lower than 97% and SNPs that had a minor allele frequency (MAF) lower than 0.05, a missing genotype higher than 0.05, or an unknown position in the rabbit reference genome (OryCun v2.0.103). The missing genotypes were imputed with the Beagle v4.1 software [18]. After quality control, 452 genotypes and 97,155 SNPs remained in the dataset. A principal component analysis (PCA) was performed to study population structure and to identify outliers using the R package SNPRelate available from Bioconductor [19].

### Identification of signatures of selection

**Fig. 1:**
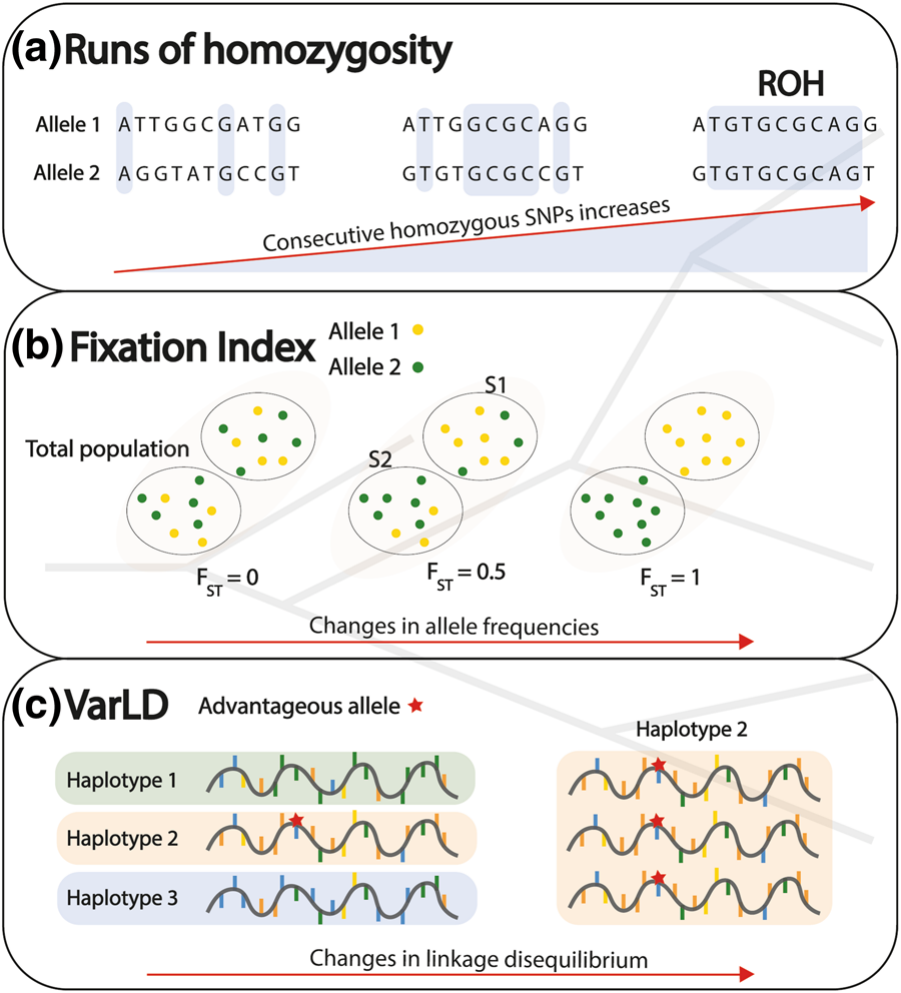
Methods of identifying patterns of signatures of selection. **a** Runs of homozygosity (ROH). From left to right: the number of consecutive homozygous SNPs increases, generating a genomic region where the individual is homozygous at all sites, i.e. a ROH. **b** Fixation index (F_ST_). From left to right: allele frequencies of individuals in the population change until it differentiates into two different sub-populations (F_ST_ = 1). **c** Variation in linkage disequilibrium (VarLD), which searches for differences in linkage disequilibrium (LD) patterns between populations. From left to right: an advantageous allele (red star) can modify the LD in a population because of the selective sweep containing the SNPs surrounding it, i.e. the haplotype of this advantageous allele (Haplotype 2)

Statistical analyses were performed to search for signatures of selection using the 274 genotypes from generation 11 (139 from the line with high V_E_ of LS and 135 from the line with low V_E_ of LS) and 90 genotypes from the base population. The 93 genotypes from generation 13 were kept for the validation analysis. Three methods were used to identify the patterns of signatures of selection (Fig. 1): (a) detection of runs of homozygosity (ROH), (b) quantification of the variation in LD patterns (VarLD), and (c) estimation of the fixation index (F_ST_).

#### Detection of runs of homozygosity

A ROH is a region of the genome that displays a local reduction of genetic variation, i.e. a genomic region for which the individual is homozygous at all sites, which indicates the presence of a locus that is affected by selection (Fig. 1a) [20]. Using an algorithm implemented in PLINK v1.9 [17], we identified the ROH in all the individuals from the base population and from the lines with high and low V_E_ of LS of generation 11. The parameters were set according to Ceballos et al. [21]. This algorithm searches for stretches of consecutive homozygous SNPs on each chromosome using sliding windows of 500 kb that contain around 50 SNPs. SNPs with missing calls and more than one heterozygous SNP were not allowed in a window. The proportion of the overlapping windows that must be called homozygous to define any given SNP as in a homozygous segment was set to 0.05%. Two SNPs separated by more than 1 Mb belonged to two different homozygous segments. A homozygous segment was considered as a ROH if the number of consecutive SNPs exceeded 50 and the SNP density was higher than one SNP per 30 kb. A ROH must be a consensus genomic region in the selected animals to be a candidate signature of selection, i.e. it had to be identified in 50% of the animals in the line with low V_E_ of LS (65), and in 50% of the animals in the line with high V_E_ of LS (70).

#### Estimation of the fixation index

The fixation index (F_ST_) was used to estimate the differences in allele frequencies between the lines with high and low V_E_ of LS (Fig. 1b). The F_ST_ was calculated using Weir and Cockerham’s pairwise estimator method [22], implemented in the VCFtools v1.16 software [23]. The F_ST_ values were estimated in 500-kb sliding windows with a step size of 250 kb. Windows with less than ten SNPs were excluded from the analysis. F_ST_ values were weighted to take differences in sample sizes between populations into account (for further details see Weir and Cockerham [22]). Relevant F_ST_ windows were those with a weighted F_ST_ value equal or above the weighted F_ST_ value in the 99.9th percentile of the distribution for all the genomes. MAF was calculated in the base population and the lines with high and low V_E_ of LS for the relevant F_ST_ windows. Those that showed divergent changes in MAF between the rabbit lines relative to the base population were considered to be putative signatures of selection. These windows were considered as resulting from an effect of genetic drift if the MAF between the lines with high and low V_E_ of LS at generation 11 displayed the same change relative to the base population (increase or decrease) or if one of the lines did not show any change (i.e. had a MAF equal to that in the base population).

#### Quantification of VarLD scores

We used the VarLD software [24] to evaluate the magnitude of the differences in LD patterns (Fig. 1c) between two populations. We analysed the pairwise comparison of the three populations: base population with the line with high V_E_ of LS (Base-High), base population with the line with low V_E_ of LS (Base-Low), and between the lines with high and low V_E_ of LS (High–Low). Sliding windows of 50 SNPs with a step size of one SNP were used to calculate the correlation matrix of each population per chromosome. The program computed the r2 metric for each pair of SNPs to determine the strength of LD in each window. The difference between the eigenvalues of the correlation matrices of both populations determined the VarLD score, which was standardized by the mean and the standard deviation of all the scores along each chromosome. A genomic window was relevant when its standardized VarLD scores were equal or above the standardized VarLD score in the 99.9th percentile distribution for all the genomes. The relevant windows identified in both the Base-High and Base-Low comparisons were considered as putative signatures of selection and the relevant windows identified only in the High–Low comparison were considered as resulting from the effect of gene drift.

### Validation

The putative signatures of selection were validated by identifying those detected in the animals of the base population and of generation 13 (45 from the line with a high V_E_ of LS and 48 from the line with a low V_E_ of LS) applying the methods described above (Section on “Identification of signatures of selection”). Only those that were detected in both analyses (i.e. in generations 11 and 13) were considered as true signatures of selection.

### Identification of candidate genes

Candidate genes were detected by searching for functional mutations in the genomic regions considered as true signatures of selection. Functional mutations were identified using whole-genome sequencing (WGS) data from two pools of DNA from breeding males in generation 10, i.e. all the fathers of animals from generation 11. Pools of DNA were prepared for each rabbit line (27 animals per line) and sequenced by Illumina Technology with an average depth of 27×.

WGS data were pre-processed following Elston et al. [25] with the following steps: (1) indexation to the reference genome (OryCun v2.0.103), (2) removal of adapters and low-quality read ends, (3) alignment to OryCun v2.0.103, and (4) identification of duplicates. Then, variant calling was performed using the GATK Best Practices pipeline [26] in three steps: (5) creation of raw VCF files for the high and low V_E_ of LS lines, (6) variant filtering, and (7) variant annotation (for further information see Casto-Rebollo et al. [11]).

A variant was considered as a functional mutation if it affected the transcription unit of a gene, i.e. (a) if it was located in the UTR regions, (b) if it was a missense or frameshift mutation, or (c) if it affected a splicing site. The gene ontologies (GO) of each candidate gene were extracted using the biomaRt package available from Bioconductor to R [27]. The Ensembl 103 release database [28] was used to access the *Oryctolagus cuniculus* v2.0.103 information.

## Results

**Fig. 2:**
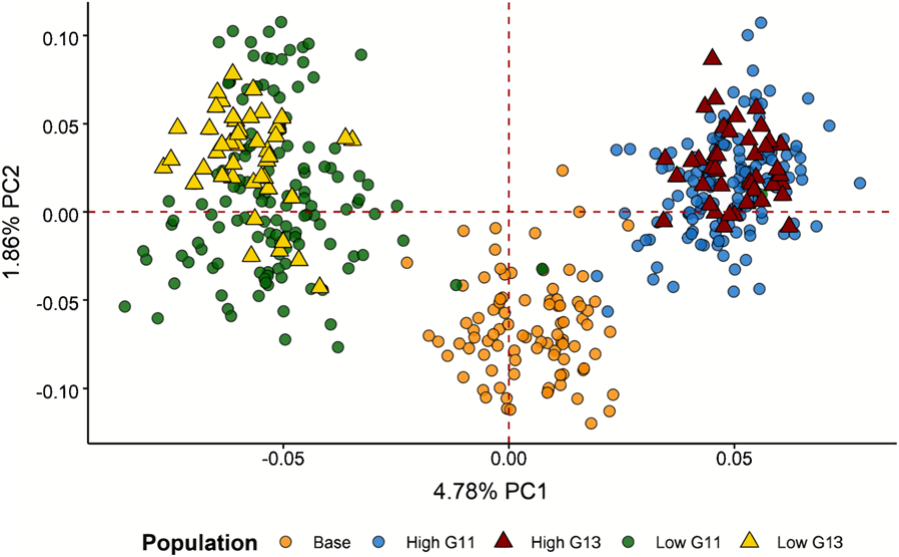
Principal component analysis of the genotyped data. Representation of the first (PC1) and second principal component (PC2) of the genotype data from the base population (orange) and the lines with high (right) and low (left) VE of LS in generations 11 (dot) and 13 (triangle)

Analysis of the population structure using principal component analysis showed a clear separation between the base population and the lines with high and low V_E_ of LS (Fig. 2). The individuals from generation 13 displayed the same family structure than that of their ancestors from generation 11.

### Signatures of selection for V_E_ of LS

The ROH, F_ST_ and VarLD methods (Fig. 1) identified putative signatures of selection for V_E_ of LS, using the animals from generation 11 and the base population. Analysis of the contiguous homozygous segments identified 6230 consensus ROH, which were detected in at least two animals of the base population and of the lines with high and low V_E_ of LS. Of these 6230 consensus ROH, 720 were identified in at least 70 does of the line with high V_E_ of LS and 65 does of the line with low V_E_ of LS. These 720 consensus ROH were considered as putative signatures of selection because they were detected in at least 50% of the animals of each rabbit line. The F_ST_ analysis identified eight genomic regions with a weighted F_ST_ value equal or above 0.35 (99.9th percentile) on *Oryctolagus cuniculus* chromosome (OCU)2 (104.5–105 Mb), OCU9 (89–89.75 Mb), OCU12 (8.75–9.5 Mb), OCU14 (121–121.5 Mb), and OCUX (81–81.75 Mb) (see Additional file 1). However, only the regions on OCU2 (104.5–105 Mb), OCU12 (8.75–9.5 Mb) and OCUX (81–81.75 Mb) were considered as signatures of selection for V_E_ of LS because they showed consistent divergent changes in MAF between the lines with high and low V_E_ of LS relative to the base population (see Additional file 1). The VarLD analysis identified three genomic regions that showed differences in LD patterns and overlapped between the lines with high and low V_E_ of LS on OCU13 (89.31–90.54 Mb), OCU14 (0.014–2.27 Mb), and OCU17 (28.78–29.92 Mb). The highest VarLD scores, 12.55 and 12.25, were obtained for OCU14 in the Base-High and Base-Low comparison, respectively. These three genomic regions were proposed as putative signatures of selection because their LD patterns differed relative to the base population.

The 726 putative signatures of selection identified in the ROH, F_ST_ and VarLD analyses were validated in 93 animals from generation 13. Among these 726 putative signatures of selection, 134 (see Additional file 2) were considered as true signatures of selection (Fig. 1), i.e. 129 ROH based on patterns of homozygous segments, two VarLD regions based on differences in LD patterns on OCU13 (89.31–90.54 Mb) and OCU14 (0.014–2.27 Mb), and three F_ST_ regions based on changes in allele frequencies on OCU2 (104.5–105 Mb), OCU12 (8.75–9.5 Mb) and OCUX (81–81.75 Mb). Finally, among these 134 true signatures of selection, the genomic regions did not overlap among the three methods.

### Candidate genes for V_E_ of LS

Nine hundred genes were identified in the genomic regions with positive selection patterns for V_E_ of LS. Candidate genes were identified by searching for functional mutations in the 900 genes using WGS data. In total, 212,845 variants (single-nucleotide variants (SNVs) and insertion/deletion variants (INDEL) were identified in the true signatures of selection (Table 1). Among these 212,845 variants, 1196 were relevant (207 INDEL and 989 SNVs) based on their location in the transcription unit of 373 of the 900 identified genes. These 373 genes (proposed as candidate genes) are involved in many biological processes (see Additional file 3). Most of them (237) are found in basic common gene ontologies (GO) such as: protein binding (GO:0005515), cytoplasm (GO:0005737) and nucleus (GO:0005634), although, they also have a pleiotropic effect on other biological processes (see Additional file 3). One hundred and eleven genes (29.76%) are involved in biological processes related to immune response (e.g., GO:0030335 and GO:0071356), stress response (GO:0042594), reproduction and embryo development (GO:0001701), and/or carbohydrate and lipid metabolism (e.g., GO:0005739 and GO:0055114).

**Table 1:** Effects of the variants (SNVs and INDEL) identified in the genomic regions with true signatures of selection for V_E_ of LS

Effect	Total
Upstream	10,925
Downstream	10,309
Intergenic	122,789
3ʹUTR	434
5ʹUTR	170
Intron	81,536
Splicing	156
Synonymous	657
Missense	368
Frameshift	38
Inframe	20
ncRNA exon	443
Stop gained	8
Start lost	2

A variant can affect more than one gene because they can share their DNA sequence, thus although the total number of variants identified was equal to 212,845, a total of 227,855 effects were found

We highlighted the *GATA3*, *FKBP10*, *KAT2A*, *CYP1B1*, *BRCA1*, *PGM3*, and *ACE* genes that have a pleiotropic effect on the immune system, lipid and carbohydrate metabolism, and reproduction and embryo development.

Among the 1196 functional mutations, we found 10 INDEL that were fixed (1/1) in one of the rabbit lines and absent (0/0) in the other and that affect the *TTC23L*, *FBXL20*, *GHDC*, *ENSOCUG00000031631*, *SLC18A1*, *CD300LG*, *MC2R*, and *ENSOCUG00000006264* genes (Table 2). These genes are involved in biological processes related to stress response (*MC2R*), energy, carbohydrate, and lipid metabolism (*ENSOCUG00000006264* and *MC2R*), nervous system (*MC2R, SLC18A1*, and *FBXL20*), immune response (*ENSOCUG0000000626)*, behaviour (*FBXL20*), cell maintenance (*TTC23L* and *GHDC*), and other processes (*ENSOCUG00000031631* and *CD300LG*). For each of the *MC2R* and *ENSOCUG00000006264* genes, one of the variants was a novel INDEL in the 3ʹUTR (Table 2) based on the two alleles in the reference rabbit genome OryCun v2.0.103. These novel alleles were fixed (2/2) in the line with high V_E_ of LS and absent in the line with low V_E_ of LS (Table 2).

**Table 2:** Functional mutations (INDEL or SNVs) fixed in one of the rabbit lines and absent in the other line

OCU^a^	bp^b^	Low^c^	High^d^	Region	Gene	Mutation^h^
9	48,280,960	1/1	2/2	3ʹUTR	*MC2R*	3-bp deletion^i^
11	56,199,847	0/0^e^	1/1^f^	Frameshift	*TTC23L*	2-bp deletion
12	138,684,799	1/1	0/0	Frameshift	*ENSOCUG00000031631*	2-bp deletion
12	138,685,173	1/1	0/0	Frameshift	*ENSOCUG00000031631*	1-bp insertion
12	138,685,179	1/1	0/0	Frameshift	*ENSOCUG00000031631*	2-bp deletion
14	33,195,918	1/1	2/2^g^	3ʹUTR	*ENSOCUG00000006264*	25-bp deletion^i^
15	4,723,998	1/1	0/0	3ʹUTR	*SLC18A1*	4-bp deletion
19	40,686,575	0/0	1/1	5ʹUTR	*FBXL20*	90-bp insertion
19	43,006,505	0/0	1/1	Frameshift	*GHDC*	2-bp deletion
19	44,276,359	1/1	0/0	3ʹUTR	*CD300LG*	8-bp deletion

^a^*Oryctolagus cuniculus* (OCU) chromosome

^b^Functional mutation location in base pairs

^c^Genotype of line with low V_E_ of LS

^d^Genotype of line with high V_E_ of LS

^e^0/0 indicates that the functional mutation is the homozygous for the reference allele

^f^1/1 indicates that the functional mutation is homozygous for the alternative allele

^g^2/2 indicates that the functional mutation is homozygous for a new allele not present in the reference genome

^h^All INDELs were marked according to the reference allele of OryCun v2.0.103

^i^With reference to the alternative allele of OryCun v2.0.103

## Discussion

Divergent lines provide good biological material for genomic studies since they are selected for a unique trait and share the same environment. Previous studies on intramuscular fat (IMF) in rabbits and pigs [16, 29], and on antibody response and feather pecking behaviour in chickens [30, 31], using divergently selected lines, successfully detected signatures of selection, and identified associated genomic regions. The principal component analysis based on genotype data in our study showed a clear separation between the two divergent rabbit lines (Fig. 2), which agreed with the remarkable phenotypic differentiation in V_E_ of LS (4.5% from the mean in the base population; Blasco et al. [4]). Thus, we searched for signatures of selection for V_E_ of LS, by combining the ROH, VarLD and F_ST_ methods (Fig. 1) to identify genes and pathways that were modified during 13 generations of selection.

In the analysis of signatures of selection, the identification of genomic regions under positive selection depends on the method applied [14]. Using the ROH, F_ST_ and VarLD methods, we identified 134 true signatures of selection for V_E_ of LS with no overlapping between methods. Indeed, as each method is based on different assumptions (Fig. 1), correlations between results are low [15, 16], which makes it difficult to detect overlaps between the identified signatures of selection. The methods used to detect signatures of selection should be considered independently of the sources of QTL detection. By combining the three methods, ROH, F_ST_ and VarLD, we were able to identify most of the selection forces that affect the trait of interest, and we considered these 134 true signatures of selection as independent patterns of positive selection for V_E_ of LS. However, only one F_ST_ window on OCU3 (51–51.75 Mb) agreed with a variance-controlling locus (vQTL), which was previously identified in a GWAS [11] that used the same animals from the base and the generation 11 populations. The three genes (*SLIT3*, *FOXI1*, and *FGF18*) with functional mutations located in this vQTL could be the most relevant genes that play a role in the control of V_E_ of LS. They are involved in biological processes related to immune response, stress response, and/or development of sensory structures, which are relevant pathways to modulate resilience [7]. However, we identified this signature of selection in animals from generation 13 using a less conservative threshold of 99.5th percentile (weighted F_ST_ of 0.37). This F_ST_ window showed a weighted F_ST_ of 0.21 at generation 11 and 0.39 at generation 13. This stronger differentiation in allele frequencies in the population from generation 13 highlights the importance of this region for V_E_ of LS. However, the difference between the weighted F_ST_ at generations 11 and 13 could also be an effect of the reduced sample size (95) at generation 13, which may hide the true changes in allele frequencies between the generations.

Previous studies in divergent populations showed that some overlapping occurred between the signatures of selection obtained by F_ST_ analysis and a few QTL identified by GWAS [16, 29–31]. In contrast to our study, those studies used populations from a long-term divergent selection (during 40 generations) [30, 31], or from a selection for a highly heritable trait (intramuscular fat; IMF) [16, 29]. The fact that V_E_ of LS has a low heritability and accuracy [12] could hinder the identification of the genomic regions under selection in GWAS. Moreover, the small or moderate size of the effect of the variants could produce a sweep that is not large or strong enough to be detected as a signature of selection [32].

The identification of relevant loci for complex traits results in a large number of candidate genes due to their polygenic nature [33]. These genes are usually involved in multiple pathways or biological processes that may not be interrelated, such that searching for a relationship between these and the trait of interest is challenging. Along the same line, WGS analysis could be useful to identify the most relevant candidate genes that underlie the complex traits under study. In this study, we identified 900 genes that spanned genomic regions with patterns of signatures of selection for V_E_ of LS, and among these, 373 presented functional mutations that affect their transcription unit (see Additional files 4 and 5). However, given these 373 genes that are implicated in a wide range of functional categories (see Additional file 6), it remains difficult to identify the most relevant molecular mechanism involved in V_E_ of LS. Moreover, these genes may not have a clear relationship with V_E_ of LS since they may be acting indirectly, by modulating the core genes underlying V_E_ of LS [34]. For this reason, we could only make hypotheses based on the genes directly involved in previously identified biological pathways for V_E_ of LS.

Previous studies, developed by Argente et al. [8, 35] and Beloumi et al. [8] on the same rabbit lines as those used in this study, showed line-differences in immune response biomarkers (plasma cortisol, leukocytes, and acute-phase protein levels), in plasma concentrations of cholesterol and triglycerides, and in mortality [8, 35]. Among the 373 genes, 59 were related to immune response, six to stress response, and 49 to energy metabolism, carbohydrate metabolism or lipid metabolism (see Additional file 3), which could explain the differences reported by Argente et al. [8] and Beloumi et al. [36]. In addition, we found 38 genes involved in reproduction and embryo development (see Additional file 3) that could clarify the correlated response of V_E_ of LS with embryo implantation, embryo survival and litter size traits [36, 37]. In our study, we highlighted the genes, *GATA3*, *FKBP10*, *KAT2A*, *CYP1B1*, *BRCA1*, *PGM3*, and *ACE*, because they have a pleiotropic effect (see Additional file 3). The ontologies of these genes are related to the immune system, lipid and carbohydrate metabolism, and reproduction and embryo development, supporting all the previously reported evidence [8, 35–37].

By searching for the most relevant functional mutations, we found seven promising genes that contained an INDEL with the alternative allele fixed in one rabbit line and absent in the other (Table 2), i.e., *TTC23L*, *FBXL20*, *GHDC*, *ENSOCUG00000031631*, *SLC18A1*, *CD300LG*, *MC2R*, and *ENSOCUG00000006264*, and which are the most relevant for V_E_ of LS. However, the functional mutation detected in the *MC2R* and *ENSOCUG00000006264* genes were even more interesting (Table 2). In our rabbit lines, both genes have lost the reference allele present in the rabbit reference genome OryCun v2.0.103 and display two different variants of the INDEL in their 3ʹUTR region. In both genes, one of the INDEL variant (the alternative variant for the reference genome) was fixed in the line with a high V_E_ of LS. The other (a new variant) was fixed in the line with a low V_E_ of LS (Table 2). The *MC2R* gene encodes the adrenocorticotropic hormone (ACTH) receptor, which controls ACTH and the level of cortisol [38]. Cortisol is an important molecule that regulates fat metabolism to mobilize glucose and mediates stress response and inflammatory response [39]. *ENSOCUG00000006264* is an orthologue of the *retinol binding protein 1* (*RBP1*) gene that is involved in the homeostasis of retinoid acid (RA) and the regulation of the vitamin A metabolism. Retinoid acid could be involved in many immunological functions, such as the control of inflammatory and tolerogenic immune response [40].

When comparing the results with previously identified V_E_ loci (vQTL), we found evidence of the implication of the immune system in V_E_, in line with the results reported by Argente et al. [8] and Belloumi et al. [35]. In their GWAS, they identified genes that are related to the triggering of inflammatory response [9, 11] and belong to the HSP (heat shock protein) gene family [10, 11, 41], which can also modulate stress response, inflammatory response as well as the levels of glucose and fatty acids [42], and the fertilization and preimplantation of embryos [43]. Elevated levels of cortisol induce the synthesis of HSP to trigger stress response and cellular adaptation [44–46]. The INDEL variant on the *MC2R* gene could affect the stability of the transcribed mRNA, affecting the expression of the ACTH receptor that modulates the cortisol response. Thus, differential cortisol response could affect the stress and inflammatory response of animals, supporting the evidence of an effect on animal resilience [8, 35–37].

Animals can maintain their performance (be more resilient) when they can discriminate environmental stimuli from background. Berghof et al. [5] proposed V_E_ as a measure of animal resilience, while Argente et al. [8] showed that the line with a low V_E_ of LS was more resilient than the line with a high V_E_ of LS. According to Colditz and Hine [7], the immune system, cell receptors, and nervous and sensory structures are essential for coping with environmental disturbances. In this study, we identified 59 genes that are involved in the immune system, 23 in sensory perception, six in animal behaviour and 35 in the nervous system (see Additional file 3), which support the correlated response of V_E_ of LS in rabbit resilience [8]. Among these 59 genes, *SLC18A1*, *FBXL20,* and again *MC2R* were highlighted because they had also GO related to the modulation of the nervous system and/or behaviour (see Additional file 6). However, the role of the highlighted candidate genes in controlling V_E_ of LS and animal resilience requires further studies to investigate their direct effect on the V_E_ of LS. Although the 373 identified genes with functional mutations were considered as candidate genes for V_E_ of LS, their direct or indirect implication in modulating the V_E_ of LS needs to be assessed.

## Conclusions

We identified 373 candidate genes with functional mutations for V_E_ of LS in rabbits by combining independent methods of detection of signatures of selection and WGS data. These genes supported the biological pathways that were previously reported to be related to V_E_ of LS and involved in immune response, lipid and carbohydrate metabolism and stress response. These candidate genes could also explain the correlated response of the V_E_ of LS in embryo implantation, embryo survival and litter size. Two novel INDEL variants were fixed in the line with high V_E_ of LS and absent in the line with low V_E_ of LS, one in the *MC2R* gene and one in the *ENSOCUG00000006264* gene. These promising functional mutations are located in genes that are involved in stress response and in the retinoid acid biosynthetic process, which could also control the immune response, respectively. This study expands on a previous GWAS for V_E_ of LS in rabbits and identified additional molecular mechanisms and pathways for V_E_ of LS and animal resilience. However, the real implications of these genes in V_E_ of LS still need to be assessed through functional analyses.

## Supplementary Information

**Additional file 1.** Relevant F_ST_ windows with a weighted F_ST_ value higher than 0.35. Signatures of selection identified using F_ST_. For each relevant F_ST_ window, the position is indicated according to the chromosome (OCU) and location in Mb. This table summarises the minor allele frequency (MAF) for each population and the weighted F_ST_ value for each F_ST_ window.

**Additional file 2.** Localization of true signatures of selection identified with each method of detection in generation 11 and validated in generation 13.

**Additional file 3.** Classification of candidate genes in general biological processes according to their gene ontologies (GO). An in-house R script was used to group the gene ontology (GO) of the candidate genes in general biological pathways. For that, we created a dictionary for each biological pathway with their keyworks (for example, cytokine for immune system). Then, we matched these keywords with the GO description of each gene extracted from Ensembl 103 (see Additional file 6) using the package biomaRt. Each description containing the keyword was assigned to its biological pathway. Gene with GO terms for which it was difficult to assign a biological process were included in the category of “Other Processes”, for example, MutSbeta complex. All keywords were extracted from the literature. The percentage of each biological pathway was calculated as the ratio between the number of GO identified in the biological pathway (Xi) relative to the total number of GO (N); $\frac{{X}_{i}}{N}$ × 100. Using this in-house R script, we obtained a general vision of the pathways based on the GO of the candidate genes. This is an initiative named PATHionary. You can support it with your knowledge through the following link; https://forms.gle/yAt3S2JDUEzQxoLu9.

**Additional file 4.** Total number of INDEL identified in the true signatures of selection for the environmental variance of litter size. INDEL identified by WGS data analysis to be located in a UTR or splicing region or with a frameshift effect. For each variant, the position is indicated according to the chromosome (OCU) and base pair (bp) location. REF shows the allele in the reference genome (*Oryctolagus cuniculus* v2.0.103) and ALT the alternative variant identified in the rabbit lines. Low and High show the allelic distribution in each line where 0 indicates the reference allele, 1 the alternative allele, and 2 a new variant.

**Additional file 5.** Total number of SNVs identified in the true signatures of selection regions for environmental variance of litter size. SNVs identified by WGS analysis to be located in a UTR or splicing region or with a frameshift effect. For each variant, the position is indicated according to the chromosome (OCU) and base pair (bp) location. REF shows the allele in the reference genome (*Oryctolagus cuniculus* v2.0.103) and ALT the alternative variant identified in the rabbit lines. Low and High show the allelic distribution in each line where 0 indicates the reference allele, 1 the alternative allele, and 2 a new variant.

**Additional file 6.** Gene ontologies and descriptions of each candidate gene identified in the true signatures of selection, using biomaRt.

## Data Availability

Data are available upon request to the corresponding author.
